# Resilience of the Childhood Origins of Dutch Mothers’ Gender Role Attitudes

**DOI:** 10.1007/s10804-015-9217-2

**Published:** 2015-09-26

**Authors:** Justine Ruitenberg

**Affiliations:** Amsterdam Institute for Advanced Labour Studies (AIAS), University of Amsterdam, Roetersstraat 31, 1018 WB Amsterdam, The Netherlands

**Keywords:** Gender role attitude, Dutch mothers, Primary and secondary socialization, Partner, Peers

## Abstract

Based on qualitative analysis of 39 in depth interviews with Dutch mothers, this paper firstly explores if differences in primary socialization are relevant to an understanding of Dutch mothers’ current diverse (personal) gender role attitudes. Secondly, it addresses the interplay of a mother’s own gender role attitude and those of her partner and peers. The findings reveal that Dutch mothers’ current diverse gender role attitudes have origins in the transmission of different mental and verbal parental, especially maternal, symbols. Egalitarian mothers often recall a strong mother-figure, the verbal persuasion of economic independence and/or particular non-traditional upbringing matters that appealed to their logic of being financially independent. The memories of traditional/adaptive mothers rather stand out for the resignation of unpaid tasks by their own mothers, and the absence of parental encouragement to consider their professional life. The findings further indicate that in childhood originated aspects of mother’s personal gender role attitude continue to shape the character of mother’s behavior and new social relationships.

## Introduction

The Netherlands is known for its varied labour market pattern among women, although, Dutch women, especially mothers, typically work part-time (OECD 2013). In 2010, 32.4 % of Dutch mothers were not in paid work, 42.5 % worked between 12 and 24 h a week, 13.8 % worked 25–35 h a week, and 11.3 % worked 36 h or more (CBS 2011). This variation makes the Netherlands an interesting case to study the origins of women’s varied employment choices. To understand this variation most contemporary researchers recognize the importance of incorporating gender role attitudes (Cunningham et al. 2005; Hakim 2000, 2003; Hoffnung 2004). Especially differences in *personal* gender role attitudes contribute to the explanation of why women employ different employment patterns (Cloïn 2010; Marks and Houston 2002; Risman et al. 1999). Personal gender role attitudes refer to the ideal of how people prefer to share work and kin care tasks with their own partner. The main contribution of this study is to achieve a better understanding of differences in the social origins of mothers’ personal gender role attitudes and to explore possible continuities and interruptions of these attitudes by diverse social influences, and hence get a better understanding of their diverse labour market behaviour. The study’s particular interest is to explore the character of transitions from primary to secondary socialization. Is this process characterized by reproduction and re-enforcement, or by recreation and flexibility?

Labour market activity is in this study understood as an outcome of a process that consists of consecutive decision-making moments, like dependent paths: each successive step depends on previously taken decisions (Vespa 2009). Attending school, whether as a high achiever or not, taking erroneously or correctly chosen continuation courses, and then the ensuing steps taking into professional work, are all not facts that can easily adjusted or reversed. Besides being based on personal characteristics, life experiences and external structural factors, like the availability of financial support, studies and jobs, these important steps forward are all partly based on peoples’ attitudes. People have developed their attitudes through the exposure to and internalization of significant others’ behaviour, norms, values and attitudes, during childhood and later in life (Bandura 1977; Berger and Luckmann 1967; Handel 2006). This interpretation of personal attitudes corresponds with the socialization or exposure-based approach (Blunsdon and Reed 2007; Bolzendahl and Meyers 2004) which assumes that attitudes develop by experiences in childhood, during the school period (young adulthood) and in early work experiences, and are relatively, but not completely, resistant to change after that time. This study focuses on the part of mother’s attitudes that possibly lingers.

The investigation of the origins and continuity of Dutch mother’s attitudes is employed with a qualitative in-depth research of 39 interviews (conducted in 2010) with mothers living in the vicinity of Amsterdam. Some considerations of the qualitative analysis are in place, and the study must be characterised as an explorative investigation. Mothers were interviewed in their adult-life, and were asked retrospectively to look back at their parental background when they were 12 years old, and to their supportive relationships in the past. This way of questioning recognises a risk of the selectiveness of memory, and the fact that mothers’ current situations and attitudes may have influenced their answers. It is possible that people may adjust their memories to justify their present behaviour (Kroska and Elman 2009). A longitudinal qualitative study, at different points in the course of one’s life, allows this risk to be avoided.

Moreover, this study is certainly not aimed at denying the interplay between cause and effect of attitudes and behaviour. Various studies have convincingly shown that women are able to shift their attitudes through work and life experiences (Cunningham et al. 2005; Kan 2007; Steiber and Haas 2012). Jansen and Kalmijn (2000) for example showed that the relationship between labour market activity and attitudes is reciprocal. The effect of emancipatory attitudes on employment is as strong as the effect of employment on these attitudes. The focus of this study is to possibly reveal the presence of a relatively perpetual part within their attitudes. In other words: which attitudes influenced mother’s behaviour while she entered the labour market for the first time, and still shape their new social relational contexts and behaviour?

## Background of The Netherlands

For a long time, Dutch female labour participation, especially among married women, was very low. In 1960, 25 % of women (Tijdens 2006), and 7 % of married women were employed, compared to 30 % of English and 33 % of French married women (Kloek 2009). Since the 1960s, as in many other Western countries, Dutch society changed radically through processes of secularization, increased educational attainment of women, and the greater acceptance of non-familial roles for women and familial roles for men (Sullivan 2004). This rise continued throughout the following decades, resulting in one of the highest levels of female participation among the Western countries, at almost 70 % in 2009 (OECD 2013). However, most of the rise of female participation was in part-time work.

Specific national characteristics can explain the heterogeneous employment—yet dominantly part-time—pattern of Dutch women (Plantenga 2002; Tijdens 2006; Van Doorne-Huiskes and Schippers 2010). In the 1970s and 1980s, when female labour participation started to rise, there were few public childcare facilities, which forced mothers who entered the labour market to combine their paid job with care tasks at home (Plantenga 1996). In the same period, employers wanted to increase the flexibility of their workforce, for which the use of part-time work was one solution. Although at first the trade unions were reluctant to support part-time work, over the course of the 1980s they became more positive, negotiating clauses on the equal treatment of part-time workers in collective labour agreements (Kremer 2007; Visser and Hemerijck 1997). Many branches started to offer family–friendly arrangements which benefitted part-time worker for men and women (Tijdens 2006). The Working Hours Adjustment Act of 2000 served to provide an employee with the right to request a reduction in the number of work hours, which could not be dismissed by the employer unless they could prove that such a move would be detrimental to the company’s interests (Plantenga 2002; Van Doorne-Huiskes and Schippers 2010).

At the same time, Dutch institutional care arrangements, such as primary school timetables, and the quality, cost and availability of formal childcare, did/do not facilitate full-time work for mothers (Plantenga 2002). And although there is political and societal support for the dual carer/worker model—50 % of Dutch parents endorse the egalitarian ideal of parental sharing (Merens et al. 2011)—contradictory social norms are also apparent. Compared to other European countries, relatively few Dutch couples agree that both partners should contribute to the household income (Haas et al. 2006). Moreover, 63 % of Dutch people consider a working week of 2 days or less to be the ideal for mothers with children up to the age of four, and only 10 % endorse the ideal of working 4 or 5 days per week (Merens et al. 2011, p. 130).

Contrary to the era of rapid social change between 1970 and 1980, the last two decades of the Netherlands are characterized by a relatively stable cultural climate as it concerns to the ideas and daily practices of gender roles (Cloïn 2013; Portegijs 2010). The share of Dutch husbands partaking in household tasks and care has shown little progress since 1995 (Bucx 2011), and part-time work remains over the years an accommodating option for most Dutch mothers (Ruitenberg 2014a).

## Theoretical Framework

### Primary Socialization

Socialization theory focuses on the social relational context in which specific normative standards and expectations are socially transmitted. Childhood is viewed as the most important formative period in life, in which the basic structure of the individual’s social world (base-world) is built, with which it will compare all later situations (Berger and Luckmann 1967; Mannheim (1959) in Everingham et al. 2007). Berger and Luckmann (1967) explained how primary socialization continues to influence people’s life and how it takes biographical shocks to disintegrate the individual perspective of social reality which is internalized in early childhood (Berger and Luckmann 1967, p. 154). They argue that during the process of primary socialization there is no problem of identification, because there are no significant others apart from the parents. It is the parents (or caretakers) who set the rules of the game.

The child can play the game with enthusiasm or with sullen resistance. But, alas, there is no other game around. […] He internalizes it as the world, the only existent and only conceivable world, the world tout court[…]By comparison with it, all later realities are ‘artificial’ (Berger and Luckmann 1967, p. 154).

According to Bandura (1977) it is important to understand that much ‘appropriate’ behaviour is learned observationally and symbolically, since often it cannot be readily established by overt enactment, since children may face social prohibitions, lack of opportunity or physical ability. Therefore, most modelled behaviour is acquired and retained through the medium of either imaginable (in the form of visual imagery or mental associations with for example the person who was modelling the behaviour) or verbal coding, referred to as mental and verbal symbols (Bandura 1977, p. 33). After modelled activities have been transformed into images, becoming easily assessable and functioning mental and verbal symbols, these memory codes will serve as guides for performance, arising automatically and outside of the subject’s own awareness (Bandura 1977). Possible mental and verbal symbols associated with attitudes towards paid work that parents have intentionally or unintentionally ‘taught’ their children include, for example, money, joy, obligation, status, independence, social life, creativity, boredom, fatigue or paternity.

Not many studies involve the impact of childhood socialization on the impact of attitudes of adults. Some studies note that the saliency of family of origin effects on ideology constructions might diminish during adolescence as the influence of adolescents’ peers and their own life experiences becomes stronger (Davis 2007). Other studies emphasize that intrafamilial continuity is likely to become more apparent as the younger generation moves into full adult status, which includes major life transitions such as marriage, parenthood and employment (Inman-Amos et al. 1994, p. 460; Biddle et al. 1980, p. 1072).

Empirical studies have shown evidence for both bearings. Children of parents with ‘modern’ values appear toe have a more egalitarian perspective on work and family roles than children of parents with more traditional values (De Valk 2008; Van Wel and Knijn 2006). Glass et al. (1986) demonstrated that parental gender ideology, such as “Womens’ lib makes sense” and “Women should have the main say in marriage” continue to significantly predict children’s orientations after childhood. Cunningham’s (2001) analysis found that children’s ideal division of labour at home is predicted by maternal gender role attitudes. Moen et al. (1997) concluded that socialization processes for women operate mainly through verbal persuasion from their mothers, rather than through role modelling (p. 291). A recent quantitative Dutch study has showed that a mother endorses a more egalitarian ideal family life if she recalls the gender role specific parental message ‘you should work in order to be financially independent of mothers’, and a positively work orientated mother at the age of 12 years (Ruitenberg and de Beer 2014, Ruitenberg 2014b). The paternal influence should not be underestimated either. A study of Davis and Wills (2010), showed that it is fathers who are particularly influential in creating the family context in which gender attitudes are transmitted from one generation to the next. As they construct the context, while being more egalitarian (direct influence) or being traditional (moderating influence via the mother), of the socialization process.

Nonetheless, Gerson (1995) argues, based on her qualitative study, that early childhood experiences are underdetermining: generally they have a weak relationship with children’s later life choices or orientations. The interviewees of her study dealt with the mixed messages of their childhoods in a variety of ways; ambivalence, confusion, and the postponement of final decisions were among the more common responses (Gerson 1995, p. 66). Starrels (1992) demonstrates with a longitudinal study that although children’s values and opinions are shaped by their mothers, he/she was unable to identify the mechanisms how it occurs. Factors such as the effect of the time spent together or feelings of trust, understanding and respect (associational and affective solidarity) did not increase consensus (p. 101).

Clearly, the time context of last mentioned studies is different to that of the mothers addressed in this study. Over the last half of the twentieth century social research documented wide spread changes in attitudes about the appropriate social roles for men and women (Sullivan 2004), uniformly toward more gender egalitarian attitudes (Axinn et al. 2011). The parents (especially the mothers) of the respondents of this study to a certain extent already paved the way for their daughters, while opening alternative scripts of behaviour to the traditional stay-at-home option once their daughter became a mother herself. This clearly differed from the parent’s, especially their mothers, own upbringings during the 1950s and 1960s, which were still deeply implicated within the ideal of women as housewives and men as breadwinners (Kloek 2009). For the mothers of the respondents who themselves were raised during this period, the withering away of the ideal of being a housewife had not yet led to new ideals or guidance for behaviour. And thus their trials of new behaviour would have been more easily and more often acting against their parents’ norms, values and attitudes, such as was found by Gerson (1995). It is interesting to examine whether socialization processes have a distinct character in the current more stable cultural time period of the Netherlands. Even more because the baselines of the socialization process, the nature of the relationship and the division of labour among the respondents’ parents, have been certainly more diverse—with an increasing group of mothers being employed and fathers taking up family roles in various ways—compared to those in 1950s and 1960s.

The first research question of this qualitative study is to examine whether the diversity of Dutch mothers’ gender role attitudes can be understood by the internalisation of different parental mental and/or verbal symbols.

### Secondary Socialization

As a person lives on, one must learn to function in any new group or organisation (sub-world) that she or he enters. People learn not only new practices, but also new values and norms, new vocabulary, and new ways of interacting with others. The formal processes of secondary socialization persist with an essential problem: they are always determined by an earlier process of primary socialization. As such, they must deal with a pre-formed self and an already internalized world (Handel 2006; Wallace and Wolf 2006, p. 290). Compared to the internalized base-world, there will be differences and disagreements about values, norms, vocabulary and ways of interacting within and among the different sub-worlds. At many levels, contradictions between and within the disparate settings exist (Bandura 1977, p. 44; Handel 2006, p. 17). Berger and Luckmanns’ principle assumption is that the individual likes their identity being confirmed, and significant others are vital for this ongoing substantiation of their identity (Berger and Luckmann 1967, p. 170). Other scholars rather argued that throughout their lives people establish an acceptable position for themselves out of all these contradictions (Eagle 1988; Gerson 1995; Handel 2006).

Women’s orientations toward decisions about, and capacities for working and parenting are thus emergent, developmental and subject to change over time (Gerson 1995, p. 199).

Some recent studies have explored how life course events and changes in women’s labor market participation are associated with gender role attitude change. They find increases in educational attainment and full-time employment result in more egalitarian attitudes, while marriage, parenthood and reductions in paid work are associated with more traditional attitudes (Cunningham et al. 2005; Himmelweit and Sigala 2004; Kan 2007; Vespa 2009). Berrington et al. (2008) suggest that it is not entry into parenthood as such, but the change in women’s economic activity as a consequence of parenthood, which is associated with attitude change. Himmelweit and Sigala (2004) show that changes in attitudes or behavior are more likely when mothers’ labour market status is inconsistent with their attitudes towards women’s employment. Some qualitative studies also provide evidence of a complex interplay of economic and identity-driven motivations for mothers’ labor market participation (Himmelweit and Sigala 2004).

Berger and Luckmann (1967) already acknowledged that partial transformations of identity are common, especially in relation to individuals’ social mobility and occupational training (Berger and Luckmann 1967, p. 181). Nonetheless, they also emphasised that even within partial transformations there is a continuing association with symbols, persons and groups who were previously significant. Due to the fact that prior associations continue to linger in people’s minds (often also physically in their lives), they are likely to protest at overly fanciful re-interpretations of people’s new identities. (Berger and Luckmann 1967, p. 181).

The second question of this study concerns whether mothers tend to sustain their (acquired) attitudes through secondary social relations, by creating and recreating the familiar, or whether they reset their attitudes if confronted with different situations including possibilities or constraints, and new models of behaviour and attitudes. Within the limited context of this paper, I only explore the agreement or disagreement with a mother’s gender role attitudes and those of her partner (or ex-partner) and peers.

#### Partner

Previous research has shown that gender-role attitudes between partners are often similar, based on homogamy in mate selections: people seek marital partners with similar (gender) values and attitudes (Inman-Amos et al. 1994). Regarding employment decisions of mothers, Vlasblom and Schippers (2005) showed that these are made within the family, and that the views of both partners concerning the division of care play an important role (also Geist 2005). Hoffnung (2004) revealed with her longitudinal study (1994–2009) among approximately 200 women living in the US, that career-oriented, ‘have it all’ women often have found partners who supported their full-time work. Their ‘husbands’ appeared more family-oriented and less career-oriented than the partners of traditional women, or they had a lower educational level and thus earning capacity (Hoffnung and Williams 2013; also Geist 2005).

According to the psychological theory of interdependence (Kelley and Thibaut 1978), partners will sometimes modify attitudes or behaviours to bring them in line with their spouses’ preferences, rather than their own (in Hochschild and Machung 1989). Empirical studies among American and Dutch couples provide contradictory evidence for which partner’s attitudes are more influential than the wife’s or the husband’s original attitudes (Johnson and Huston 1998; Kalmijn 2005). Kalmijn showed that especially women’s prenatal gender role attitudes have an indirect effect on men’s attitudes through her change in employment after the birth of their child (Kalmijn 2005). Decisions about how to combine working and caring are thus still seen as women’s business. In addition, Schober (2013) found that that higher absolute wages and more egalitarian attitudes of women before motherhood reduce the shift towards a more traditional division of labour after couples have their first child. Putting it differently, women’s prenatal attitudes linger not only in her own postnatal attitudes but of also in those of her partner. However, the relationship depends on the socio-cultural context (Schober 2013). The conclusion that women’s egalitarianism reduces the change towards a more traditional division of labor after the birth of a child is consistent with other recent research employed in Australia and Britain. Nonetheless, earlier American studies found smaller effects and suggest that fathers’ attitudes are more important.

These variations may suggest that more generous family–friendly provisions for mothers in the United Kingdom compared to the United States provide women with more choice to follow their attitudes or historically embedded gender norms, which maintain that mother care is best for young children (Schober 2013).

Continuing on previous empirical research, this research explores how the dynamics between the gender role attitudes of mother’s with her (ex) partner resemble the parental origins of a mother’s own gender role attitudes?

#### Peers

Socialization among peers is conceptualised by Ryan (2001) as a process that occurs through frequent interactions, shared experiences and exchanged information among a relatively intimate group of friends who interact with each other on a regular basis (Ryan 2001, p. 1138). Much relevant research has been done on adolescents peer groups (e.g. Biddle et al. 1980; Grusec and Hastings 2007), and it is widely recognised that adolescent peer relationships or peer group pressures have consequences for emotional adjustment, school achievement, and risk-taking behaviours (Biddle et al. 1980; Windle 1994). Concerning employment behaviour among adults, several studies have shown that labour market behaviour can be modified by the behavioural example of other people in the environment, and that other people can act as role models (Sealy and Singh 2009). Some social-psychological studies have shown that someone’s labour market behaviour can be modified by the behaviour of important people in his or her environment. Woittiez and Kapteyn (1986), for example, demonstrated the existence of a so called ‘bandwagon effect’ among Dutch women in a social group. This effect refers to the fact that if a member of a social group enters the labour market, his or her entrance motivates other members of the same social group to join the labour market as well. This theory corresponds with sociological notions of role models (Bandura 1977). A role model can be an inspiring and motivating person, someone from whom one can learn, providing a script for behaviour in particular contexts (Sealy and Singh 2009). Portegijs et al. (2008) has found a significant impact on the participation level of mothers if other mothers in their environment work or make use of formal childcare. Also Blaffer Hrdy (2000) concluded that the expectations that mothers have of their own lives are based on their own ideas and on the ideas of how it should be by others.

However, one should realise that in general people choose friends with similar ideas, attitudes, interests or characteristics as themselves. Or, as Brown et al. (1993) argue, people do not haphazardly fall into one crowd or another; similarities are prevalent a priori to relationships. The continuum of mother’s own gender role attitudes and those of her friends is of particular interest within this paper.

## Research Method

In order to answer the research questions, semi-structured face-to-face interviews have been conducted with 39 mothers, all of whom having at least one child younger than 12 years old living at home, and all living in the vicinity of Amsterdam, The Netherlands. The age at which most parents deem their children old enough to be left on their own is 11 (Duncan 2005). The interviews took place between April 2010 and November 2010. The interviews took on average one and a half hours to complete, and full transcripts of the interviews were made. In order to select the interviewees, four categories of mothers were differentiated according to their employment patterns: mothers who work 0 h (referred to as stay-at-home mothers or full-time homemakers), 12–24 h a week (mothers with a small part-time job), 25–35 h (mothers with a large part-time job) and 36 h or more (full-time working mothers).

As is well known, higher educational levels lead to higher levels of labour participation, especially among mothers (Merens et al. 2011). Sufficient education is then understood as a precondition for labour market participation. For example, higher educated women work more, because their higher wage allows them to pay for child-care facilities. They may also have been exposed more to critical ideas and formed career-oriented networks (Cunningham et al. 2005; Doorewaard et al. 2004, p. 11). In 2009, 37 % of Dutch higher-educated mothers worked more than 35 h per week, compared to only 18 % of lower-educated mothers. 52 % of lower-educated mothers did not participate in the labour market at all, as compared to 12 % of higher-educated mothers (Central Bureau of Statistics 2011). In each of the four employment categories, there were approximately equal numbers of lower- (intermediate vocational level and lower) and higher-educated mothers (higher vocational level and university).

For theoretical reasons, the sample of interviewed mothers was drawn largely within one urban area, Amsterdam. In this way, differences in employment behaviour and attitudes among respondents do not differ with respect to the influence of structural and cultural factors that may diverge between urban and rural areas, such as the availability of childcare provisions, jobs and religiousness, which could also affect potential differences in gender and work attitudes.

In order to achieve good correspondence between research questions and sampling, a strategy of purposeful sampling had to be followed (Bryman 2008, pp. 458–459). To be able to fill all eight categories (four along employment patterns and two along educational levels) of mothers within one area equally, the respondents were found using the snowball method. Firstly, a small group of mothers in the social environment of the researcher, the so-called weak ties (Granovetter 1973), was approached, especially at a primary school in Amsterdam (Old West Quarter). Subsequently, the other respondents were approached on the advice of the first group of respondents. The collection of material ended when theoretical saturation was reached, and new interviewees did not bring more diversity. Quite clearly this research method cannot produce a statistically representative sample, since it relies upon the social contacts between individuals to trace additional contacts. The research method, however, does permit revealing the reciprocal character between primary and secondary socialization, and is able to highlight what mothers consider as being relevant when describing their childhood and further social relational contexts.

### Research Group

The interviewed mothers were born between 1962 and 1980. Their average age was 39.3 years. Seven interviewees (18 %) had a non-Dutch background (at least one parent born outside the Netherlands). Within the research group, 23 mothers were highly educated (higher vocational education and university), and 16 lower educated (intermediate vocational education and lower). Ten mothers were full-time homemakers, 8 mothers had a small part-time job (12–24 h), 11 mothers had a large part-time job (25–35 h) and 10 mothers worked full-time. Appendix 1 provides an overview of the backgrounds of the respondents. All names in this paper are anonymised.

### Interview Questions

The interviews can be characterised as oral life history interviews (Bryman 2008). The interviewees were invited to look back at specific moments in their lives, especially during childhood, while also concentrating on the behavioural steps of later social life, from finishing high school, choosing a continuation course, entering their first job and giving birth to their first child. Several open questions were asked in order to discover how and with which words women refer to these themes themselves.

*Personal gender role attitudes* refer to a mother’s desired division of labour with her own spouse. This personal ideal was more closely examined by mothers’ satisfaction with her current division of labour. A traditional personal gender attitude means a desire to have the main responsibility at home, while her partner is in paid work. An egalitarian personal gender attitude implies a wish to share paid and unpaid work equally. Adaptive attitudes are here defined as the personal desires to combine paid work and family tasks, with consent to the idea that mothers have more responsibilities at home and fathers may work full-time. (Do you have ideas about the ideal division of labour with your spouse? Are you satisfied with your own current division of labour? What would you like to change?). Also some questions were asked about her general ideas about the ideal division of labour between men and women. Questions towards a mother’s general gender attitude were for example: How do you perceive differences between men and women? Do you have an opinion about full-time working mothers or mothers who are not employed?

*Work attitudes* are defined as mother’s personal motivations to pursue paid work. (What are the most important reasons for you to work? Did you have ideas about your future profession at a young age?). A strong personal work attitude means that someone was already as a young adult strongly oriented to pursue paid work and is intrinsically motivated to work.

Following this, the mothers were asked several questions about their family backgrounds, looking back to when they were 12 years old. In particular, this included questions addressed the gender division of labour of their parents (Did your parents work? Did your father help with household chores and childcare? Were your parents happy about their division of tasks?), parental attitudes (What were the important norms, values, and (implicit and explicit) messages that were transmitted by your parents?). In addition, several questions were asked on upbringing matters, like whether the parents were strict or encouraged their daughters to fulfil their full potential at school or at work.

Finally, several questions were asked about how mothers perceived their attitudes and behaviours to have been influenced by their partners and close friends in fulfilling their full professional potential.

### Interview Analysis

The research was specifically sensitive to perceiving the lives of the respondents in terms of continuity and process, especially referring to the theoretically assumed continuity throughout the course of life between primary and secondary socialization processes. Therefore, the interview transcripts of each respondent were not cut into different codes, but kept as close as possible to each story told by the respondents.

The main part of the analysis consisted of searching for sensitizing concepts that could be used as pegs to describe the central narratives of, and the similarities within, the different groups. The sensitizing concepts were: acceptance towards the marital division of labour of her own mother, the nature of memories of her mother (or the mother-figure), message importance of economic independence, parental support towards their daughter’s professional life, presence or absence of stimulating partner or friends towards mother’s professional life. Following this, the transcripts were reread while focusing on these sensitizing concepts, and memos were written throughout the process. Below, the findings are described along the three research questions, while attention is given to the sensitizing concepts. The narratives of mothers’ attitudes could be patterned along two groups: traditional-adaptive attitudes versus egalitarian attitudes. The differences between these groups are specifically addressed.

## Findings

### How Can the Diversity of Dutch Mothers’ Gender Role and Work Attitudes be Understood by the Internalisation of Different Parental Mental and/or Verbal Symbols?

In this study, the interviewed mothers with more traditional attitudes had a few common characteristics, although there were also often exceptions, which are addressed if relevant. Firstly the relatively traditional mothers have no job, or else, work small part-time jobs. They tend to perceive it as their natural role to execute most of the unpaid family tasks, and do not put much value on their economic independence. They also appear mostly satisfied about the division of the work at home with their partners, which is often around 80 % of the tasks for the mothers and 20 % for the fathers. Contrary, mothers with more egalitarian gender attitudes, who often have large part-time or full-time jobs, cannot imagine not working themselves; moreover they consider it as unwise, citing the necessity of economic independence. They also expect a more equal share from their husbands in the unpaid tasks at home, which full-time working mothers have greater success in achieving.

Mothers with traditional or adaptive gender attitudes often emphasise that they come from warm families, and often have many happy memories from youth. Consistently, the relationship with their parents is still close. On the question of who is a shoulder to lean on when they must make a difficult decision, traditional or adaptive mothers often recall their parents. The parental division of work used to be traditional, not because their mothers did not work, but especially in the sense that their mothers unquestioningly did most of the unpaid family work. The presence of a caring mother, who carried out her unpaid duties without complaint, is recollected as a natural and self-evident situation by their traditional or adaptive adult daughters. Often, the interviewees have no clear memories of their mother during primary school. Yet, mostly they presume that their mothers liked their role of child carer and housewife, although they admit they never really asked their mother: “I actually don’t know. I don’t have the impression that she missed anything.” Respondents recall that their fathers pursued full-time jobs, and often also at home were also the boss.

My mother did everything. I found that normal. I cannot remember her complaining about it, or that she found it too much work or too busy. I think she enjoyed it, I have never asked her, to be honest. My father was just an authority (Astrid).

Almost half of the mothers of relatively traditional daughters worked, mostly part-time, often to assist their husbands in their store or family company, which not always was their own ‘choice’ but rather occurred from necessity. Often these mothers worked during school hours, and were at home when their children came back from school.

My mother was always there after school. She worked in the mornings. I never questioned what my mother did, when I was at school. She was always there, yet I know she had a job (Esther).

When we were older my mother had two jobs, as a general practitioner. Yet, she did most tasks at home, she was always there, she was the one who cared for us, which was so normal (Duke).

Sometimes these mothers’ return to work meant an essential change of family life, which was not always liked by their daughters. Especially if their mothers became too occupied with their jobs, their daughters could come to feel neglected. As a consequence, two daughters tend to show the opposite behaviour of their mothers, now they are mothers themselves. This is illustrated by Nora who temporarily gave up her job as a judge to care for her four children:

I thought it’s constantly about you and it’s constantly about your job. And for her, it was a huge part of her confidence; she got a lot of self-esteem from her work. I found that really stupid… I wouldn’t say I come from a warm nest, my mother was too preoccupied with her own business.

In addition a remarried stay-at-home mother, Mireille, who knows she can stand on her own legs, which she did the period after her divorce, is now happy she can afford to stay at home because her husband earnings suffice. She remembers how she pitied her mother, “after I was sixteen, I went always straight home from school, because I found it sad for my mother, because I knew she was not happy.” Nonetheless, before puberty she really liked it that her mother was there for her after school, and therefore she wants to be there for her own children when they are still young.

The parental work ethic received by daughters with now traditional or adaptive gender attitudes was: follow a good education to be able to contribute to society. The narratives of stay-at-home mothers in particular reveal that after they finished high school, their parents were not particularly helpful in assisting their daughter’s choice of continuation course or profession: “They never asked me, ‘what do you want to be, what is important for you?’”.

The family backgrounds of the interviewed mothers with *egalitarian* attitudes appear more diverse. Remarkably, the interviewees were often raised in non-standard families. Roughly one-third of the mothers with egalitarian attitudes were solely raised by their mothers, as a result of divorce or through alcoholism, disability or death of the father. Sophie, a mother of 48 years old with one child, who works 32 h as a copywriter, describes her youth with her alcoholic father.

He read nothing, didn’t have one friend, no contact with the neighbors, or anything. When we came home, he just sat there, sloshed in his chair, and every day a lot of fuss, you know, shouting in the house.

Yvette, a 42 years old carpenter with two children who grew up with 6 brothers and sisters and a single mom, describes her childhood after her father had deceased when she was 8 years old:

It was just natural that everyone did something. You saw that mom did everything and that was not right, so we helped. We got the groceries; my mother was not a very domestic mother. So we actually grew up like this: we had to take care of ourselves and of mom.

Other stories of egalitarian mothers reveal that their parents did not give them much attention when they were young. One daughter went to a boarding school in England, another daughter experienced traumatic family happenings at a young age, which preoccupied her parents, and there were parents who always fought. Additionally, the mothers describe upbringing matters that made them independent women, sometimes reluctantly. For example, some mothers were only 11 or 12 years old when they were made responsible for taking care of their parents’ shop while they were away on vacation, or for baby-sitting much younger brothers and sisters. One respondent was ‘pushed’ onto the train to Amsterdam alone (while living in Groningen, 220 km away) so that she could go and purchase her desired rucksack.

Also recognized from the narratives is the role of respondents’ own mothers, who generally were not described as self-evident and consenting mother-figures, as is the picture that emerges in the chronicles of traditional/adaptive mothers. Regularly, the respondents asserted that if their mothers had lived in the present, they certainly would have worked, or would have had a different job. The daughters often describe their mothers as being clever, assertive, full of initiative and reluctant to fulfil the mother role. Two egalitarian mothers saw their mothers as anti-examples as well, in the sense that their mothers behaved as victims of their era and complained about not having had the chance to do the profession they would have liked. It seems that such mothers’ reluctant attitudes towards the traditional mother role, and subsequent feelings of regret, has stimulated their daughters to fulfil their own work potential.

Another pattern among mothers with egalitarian attitudes is that they have explicitly or implicitly received the message (verbal symbol): “Make sure you can stand on your own two feet”, or “You must not rely on a man”. As mentioned before, it was not always necessary to spell out the message, but obvious because their mothers were sole providers.

Particularly my mother used to encourage me a lot, and I feel it is nice to have a lot of encouragement. Yet, maybe my mother encouraged me a little bit too much (Michelle).

Straight after finishing high school, I went to university, and that was really because my mother was pushing me, like ‘you should not spill a year, then you will never start studying. Later, I have regretted the fact that I went along with her, because it was not my own feeling’ (Olga).

Additionally, some mothers with egalitarian attitudes describe how they received contradictory messages, like was found in Gerson’s study (1995). They were stimulated to finish school and pursue careers, yet, they were also expected to marry at the age of 24 or have children and work 3 days and, preferably to live nearby their parents. Maybe, when mothers have received conflicting parental messages, mother’s own gender role attitudes are more receptive for the attitudes of other people, such as those of her partner?

### How Do the Gender Role Attitudes of Mother’s (Ex) Partner Resemble a Mother’s ‘Own’ Gender Role Attitudes?

Firstly, the findings indicate, as is previously found, that generally there is agreement among the partners about their marital division of labour. The narratives of mothers with rather traditional/adaptive gender attitudes reveal that the number of hours they work and kin care responsibilities are not key subjects of their marital discussion, but rather something often taken-for granted. For example, a mother’s decision about how much she works, and subsequently how the children are taken care of, appears to be mainly her own decision, with partners tending to ‘leave it’ up to their wives. This is illustrated by Leontien, a 42-year-old a highly educated stay-at-home mother with four children:

And what did your husband think about the fact that you wanted to give up your job?

*I think for him it was easy.*

He was quite happy with it?

*Ithink hethoughtitwas comfortable, yet, healways left itto me.*

Did you talk about it together?

*Yes*, *Ithink so*.

Did it feel like a mutual decision or like your decision?

*It was my decision, but shared or agreed by him. He said: If that is what you want, it*’*s fine. If I had said: I want to bring our son three days to the crèche, then he would also have said: that*’*s fine.*

Stay at-home mothers describe their partners quite often as egalitarian husbands.

He always said, and I believe him: ‘You must do what you want. If you want to work then we can arrange an au pair or bring the children to the day care. If you don’t want to work, it is fine as well’ (Nora).

This marital decision-making process vis-à-vis mothers’ employment activity expresses two rather opposite ideologies. On the one hand, it expresses the modern view that work is something personal to decide upon, but on the other hand, it might also reveal a rather traditional attitude that work for mothers is not a self-evident matter, and that not working is a viable option. Whatever attitude prevails, the partner’s apparent tolerant attitude leads to the situation that how children are taken care of mostly depends on their mother’s decisions in relation to work.

Nevertheless, the stories of stay-at-home mothers disclose that their decisions to give up work was not always such a pre-planned or positive choice for motherhood, but often the result of a sequence of unfavourable happenings. In this light, partners’ tolerant or phlegmatic attitudes allow a mother to slip into a non-working situation that does not necessarily make her life easier or happier. In addition, there are examples of husbands or partners who did not comply with earlier plans that they would work less. But this has not led to an apparent conflict between the partners, but rather mothers dealt with and adapted to the situation. Moreover, some interviewees emphasise that if their partners clearly and regularly show their appreciation of the way they execute their household tasks and kin care responsibility, they are fully content to perform their ‘duties’.

We have divided the tasks fine, he is the full-time worker. He leaves home in the morning between half past seven and a quarter to eight, and 11 h later he returns. But Walter doesn’t hit the sofa, as soon as he returns, he keeps working […] Walter will put the garbage out and Walter manages all the business stuff […] I do everything with love, although, I need to hear from him, ‘You did that really well,’ or ‘Hey, that’s done—that’s wonderful!’ (Nel).

The help from their husbands or partners is not taken-for-granted by mothers with more traditional attitudes, but is appreciated largely. The unpaid tasks are divided along recognisable gender lines, but the inequalities that come with that are unquestioned.

I think I do more, it just doesn’t feel like he’s is not the type that hits the sofa. It’s more a consequence of the fact that I am home more than he is (Carien).

Egalitarian mothers, especially those in full-time work, seem to have ‘found’ partners who are more encouraging towards their wife’s work ambitions. Moreover, often they are proud of their wife’s career and would not appreciate if she wasn’t working. These partners take up a share of the unpaid, domestic work more automatically and without much resistance, which is illustrated by the following quote.

I talk a lot with my husband about work and about my aspirations. And he encourages me, for example with my idea to go back to university […] He is also someone who always says, ‘hey, if you need to work longer, then I’ll take the kids home today’ (Annemiek).

Most mothers within egalitarian attitudes seem to practise their ideal, especially if their husbands work 4 days per week as well. These mothers realise their rather exceptional gender division of labour, and describe their partners as unmacho men, as gentlemen, or as caring fathers. The greatest contrast with the more traditional women is that for mothers in this group, it is certainly not a self-evident matter to take the lion’s share of the household work: “Preferably, I do nothing in the household” (Cathy). The mothers often emphasise that they would never be able to work that many hours without their partner. In some cases, their husbands perform the majority of the unpaid tasks. If gender roles are reversed, these are emphasised and remarked upon.

### How Do the Gender Role Attitudes of a Mother’s Peers Resemble Her Own Gender Role Attitudes?

In the stories of the interviewees, it emerges that the resemblance of close friends on the gender role attitudes of mothers seems less than expected based on research among adolescents (Ryan 2001). Mothers even hesitate to describe the gender role attitudes of their best friends. Sometimes, this was because they were reluctant to speak for other people, but other times this was because they simply did not know. This is revealed through the following quote from Sheila:

I suspect that sometimes they think ‘gee, why does she work so much?’ I think that, yes (Sheila).

A reason for this lack of knowledge is that most women identify their best friends as the ones they have known since high school or later educational years. Since their youth, mothers and their close friends have experienced a great deal together, such as graduations, weddings, funerals, and the birth of their children. At present, these old and dearest friends often do not pursue the same lifestyles as the mothers themselves, for example because these friends are without partners or children. In addition, most mothers confess that they do not see their best friends much, due to the time commitments of work and children, and sometimes because their friends live in other areas of the country or abroad. Often mothers have also made new friends, for example those they have met at their child’s school, or else at work. Although mothers are careful when describing these new friends as ‘close’, these new friends tend to have more similarities with their own current lifestyles, and ‘everyday’ contact makes it easier to discuss more everyday subjects, such as household quarrels and grievances.

The social pressure of other people in their social environment, other mothers in particular, is also discussed by the respondents. It appears that if mothers behave in line with the current Dutch norm, which implies a working week of about 3 days (Portegijs et al. 2008), then mothers’ narratives reveal how much their life style is socially accepted, as is illustrated by Nel psychologist, working 2 days and mom of a 2 year-old:

My sister in law says that I have it perfect, because I still develop myself, I am not completely out of the labour market, and at home I can also be very much present too.

But in the case of mothers who do not suit the current Dutch norm—either due to being a stay-at-home mother or working full-time—the social atmosphere is less accepting. This affects in particular mothers who do not work.

The general hip and trendy working women, you know, I feel they have their opinion ready. I often want to shout: ‘You don’t know what it’s like, woman, to have twins!’ (Janne).

Sometimes it is very difficult because you feel the pressure from outside. People ask me: ‘So what do you do all day?’ They think I drink coffee the whole day (Mireille).

Mothers with egalitarian gender attitudes seem to be less affected by the weaker social circle:

You know when you work, you are not concerned with the mothers in the schoolyard. What people think of me there has never interested me at all, really. I don’t care. No, I even refuse to be sensitive to that (Claire).

Nonetheless, also among egalitarian mothers one can perceive a pattern that they rather live in social environments that match their own norms and attitudes. If norms and values of the neighbourhood differ too much from their own, mothers (and their families) seem impelled to move away, because they did not feel at ease, of which Cathy, a mother who received contradictory parental gender role messages, who lives now with a (in her own words) very ‘unmacho’ man, working 4 days and mom of an 11-year old son, gives an example:

Yes, when we had our son, we decided to live outside Amsterdam. But there, I felt the worst mother ever. He was the only one from school who went to after-school care. He was picked up in a little van and went to another village. All the kids went home for lunch… lunch! Drama—I felt terrible (Cathy and her partner subsequently decided to move back to Amsterdam).

Hence, mothers do seem sensitive to real and supposed expectations, and to the approval of other people. Nevertheless, it is difficult to perceive whether and to what extent these social influences and subsequent feelings affect mothers’ attitudes. It seems that the ability of mothers to “resist” influences in their environment also varies according to the ‘power’ of their position. For example, working mothers felt able to ignore the judgements of mothers in the playground, or even physically move away from disapproving others. By contrast, stay-at-home mothers felt less able to resist what they perceived to be negative judgements of others. Nonetheless, the narratives do reveal that people do not haphazardly end up in matching social environments, and childhood originated origins of gender role attitudes do have a traceable resilience.

## Conclusions

Most Western mothers prefer part-time work in order to achieve a work-life balance (Jacob 2008; Fagan 2001; Reynolds 2003). Therefore the availability of relatively ‘sophisticated’ part-time work in the Netherlands, enabled by Dutch laws, policies and collective agreements at industry level, is mainly perceived as a privilege in the Netherlands as well as by other Western Countries (Wiesmann et al. 2010). Presumably, more than in other countries, Dutch women can choose whether they want to stay at home with their children, to work at a small or large part-time job, or to continue working full-time. For that reason the Netherlands seems a suitable case to explore the social origins of mothers’ diverse labour market decisions.

Various empirical Dutch studies have shown that the diversity of female employment in the Netherlands is related to women’s diverse gender and work attitudes (Beets et al.^1997^; Cloïn 2010; Hooghiemstra 2000; Kraaykamp 2012; Portegijs et al. 2008). This qualitative and explorative study sheds light on the question of whether an explanation for this diversity lies in prior socialization processes.

Firstly, the findings showed how differences in primary socialization are relevant to an understanding of Dutch mothers’ current diverse gender role attitudes. Various mechanisms and patterns emerge. It appeared that intergenerational influence mainly occurs via the transmission of mental symbols, both intentionally and unintentionally, and especially diffused by the respondent’s own mother, as demonstrated by earlier research (Moen et al. 1997). Children do not automatically mimic parental behaviour; it is a much more subtle affair (Gerson 1995; Mason 2000, p. 240; Moen et al. 1997). Parents’ implicit messages, attitudes and feelings concerning their own division of labour are picked up by their offspring, such as feelings of unfairness about the actual division of labour or regrets about missed opportunities.

Examples of the transmission of mental codes are the respondents’ mothers’ own attitudes towards the traditional mother role, like their being consenting or reluctant with this role. The interviewees with traditional/adaptive gender attitudes seemed almost unquestionably familiarized with the silent presence and submissive, receptive performance of unpaid tasks by their own mothers. Now being mothers themselves, they naturally and automatically identify with the traditional ‘mother role’. The narratives of mothers with egalitarian attitudes stood out due to their strong memories of their own mother’s presence. She could be the dominant sole provider, or else could be unsatisfied with the mother-role. There were also few anti examples where the maternal codes were too penetrating and in cases even irritated their daughters, causing their daughters to go on and develop opposite attitudes.

The findings further illustrate that it is relevant to notice those thing that were absent from the narratives of mothers’ childhoods. For example, there may have lacked a specific message relating to their daughter’s future profession. When mothers have not been stimulated (either verbally or mentally) to consider their professional life seriously, it seemed difficult to overcome this arrearage later in life. As expected, a clear parental message towards work and financial autonomy appeared more present in the childhood stories of mothers with egalitarian attitudes, and more absent in the recollections of traditional/adaptive mothers, playing a lucid self-determining role later in life. Additionally, some specific and unusual family situations called upon daughters to become responsible at a very young age. The interviewees who now support symmetrical gender roles often seemed in different ways more or less ‘forced’ to grow up as independent young women very quickly—a childhood characteristic resulting in the appearance that they are well-equipped for the labour market.

With respect to processes of secondary socialization, it seems unlikely that the part of personal gender and work attitudes with origins in childhood may easily adjust to changing circumstances. This study confirms that in general, people seek and marry partners with a similar sex-role ideology as their own (Inman-Amos et al. 1994). Egalitarian mothers often have found ‘fitting’ partners who are stimulating towards their career ambitions. This is contrary to the conjugal discussions within traditional/adaptive couples, where it appeared not automatically evident that mothers work (also Moen et al. 1997, p. 587).

Guided by one’s sympathies and antipathies, affections and aversions, tastes and distastes, one makes for oneself an environment in which one feels ‘at home’… And we do indeed observe… a striking agreement between the characteristics of agent’s dispositions… and those of the objects with which they surround themselves (Bourdieu 2000, p. 150).

The impact of peer groups on mothers’ gender and work attitudes remained underdetermined. The interviewed mothers generally did not belong to homogenous peer groups. Nonetheless, anonymous people ‘out there’ seemed, at least in the mind of some interviewees, able to pressurise mothers’ feelings, especially by stay-at-home mothers who generally feel undervalued by society for the work they are doing (also Zimmerman 2000). Some stories of egalitarian mothers of which most of the have large jobs, symbolising a more powerful position in society, disclosed that ‘traditional’ social environments were reasons for them to move to other neighbourhoods, where they were more surrounded by people with similar gender attitudes. This mechanism illustrates that people do not easily adjust their attitudes, but rather find ways to reconcile them.

Recollecting the central question of this study, the findings revealed that Dutch mothers’ diverse current gender and work attitudes did not arise from nowhere, but are (at least partly) grounded in childhood experiences. Later situations and social interactions can modify the intensity of these attitudes, yet some important aspects of attitudes that are originated in childhood—transmitted by parental mental and verbal symbols—seem resistant to change, and rather—often automatically and unintentionally—appear to be re-enforced.

Obviously, this study has an explorative character, and more qualitative research on the micro-interactional processes would help to understand and reveal the construction, reconstruction and consolidation of people’s social realities. Moreover, there are good reasons to believe that if we want to understand the way people shape their networks and environments in order to match their attitudes, research on the ‘enduring’ influence of parents on their adult children’s attitudes remains relevant. Several researchers of intergenerational ties argue that family-ties between parents and their offspring have not been weakened but strengthened in the past decades. This increased interdependence is explained by increasing longevity, augmented the number of years of shared lives between generations, the drop in fertility rates which has increased the bonds across generations (Van Gaalen and Dykstra 2006). Moreover, under the influence of individualization, family relationships are becoming more like achieved ties. Given these socio-demographic changes, Bengston (2001) predicts a larger significance of intergenerational bonds in the twenty-oneth century in all Western societies (in Van Gaalen and Dykstra 2006, p. 947). Consequently, transmissions of attitudes and behaviour across generations remain relevant in studies on labour market behaviour.

## Appendix 1: Overview of Respondents

**Table:**

Name	Age	Work–hours	Work hours partner	Number of children	Age of children	Education	(Former) profession	Gender attitude
Janne	38	0	40+	3	4, 4, 9	Higher polytechnic	Physiotherapist	Traditional/adaptive
Leontien	42	0	40+	4	6, 8, 10, 12	University	Shop employee	Traditional/adaptive
Nora	40	0	50	4	2, 5, 5, 7	University	Judge	Traditional/adaptive
Barbara	39	0	40	4	3, 5, 8, 10	University	Recruiter	Traditional/adaptive
Astrid	43	0	36	3	8, 5, 3	Higher polytechnic	Controller	Adaptive
Marieke	42	0	–	4	13, 10, 9, 6	Intermediate polytechnic	Cashier	Traditional/adaptive
Mireille	35	0	40	3	13, 7, 5	Secondary school	Personal assistant	Traditional/adaptive
Nienke	47	0	40	1	8	Secondary school	Manager	Egalitarian
Meriam (Iraq)	48	0	Unemployed because of disability	3	25, 22, 8	Primary school	None	Traditional/adaptive
Saida (Turkey)	33	0	30	3	8, 4, 2	Primary school	None	Traditional/adaptive
Heleen	34	24	40	3	7, 4, 1	University	Teacher	Traditional/adaptive
Nel	32	16	40	1	2	University	Psychologist	Traditional/adaptive
Tineke	38	24	32	2	13, 3	Intermediate polytechnic	Professional child care	Traditional/adaptive
Youssra	35	18	Unemployed	3	10, 8, 6	Primary school	Cleaner	Traditional/adaptive
Brigitte	35	24	40	2	5, 1	Intermediate polytechnic	Professional child carer	Traditional/adaptive
Carien	35	24	40	1	1	Intermediate polytechnic	Project assistant	Egalitarian
Willemien	47	24	Unemployed because of disability	1	8	Intermediate polytechnic	Cook	Traditional/adaptive
Esmé	34	24	40	2	4, 2	Secundary school	Personal assistant	Traditional/adaptive
Juul	40	32	32	2	2, 10 Months	University	Publisher	Egalitarian
Medina	38	32	32	3	5, 4, 11 Months	University	Prosecutor	Egalitarian
Alice	47	30	40	2	13, 10	Higher polytechnic	Journalist	Adaptive/egalitarian
Marloes	43	30	40	2	11, 8	University	Translator	Adaptive/egalitarian
Cathy	44	32	32	1	11	University	Manager	Egalitarian
Diana	42	30	–	2	10, 7	University	Psychologist	Adaptive/egalitarian
Sietske	43	32	40	3	11, 8, 8	University	Director	Adaptive/egalitarian
Olga	30	28	32	2	11 Months	University	Social worker	Egalitarian
Yvette	42	30	40	2	7, 6	Intermediate polytechnic	Cabinet	Adaptive
Sophie	48	30	36	1	9	Intermediate polytechnic	Copy writer	Egalitarian
Annemiek	37	34	34	2	7, 5	University	Policy maker	Egalitarian
Lotte	47	40	40	2	7, 9	University	Manager	Egalitarian
Michelle	47	40	–	2	13, 9	University	Managing director	Egalitarian
Ilse	43	40+	40	3	12, 10, 5	University	Publisher	Egalitarian
Marlieke	47	36	Variable	2	11, 8	University	Policy maker	Egalitarian
Claire	47	36	36	2	12, 10	University	Managing director	Egalitarian
Ebru (Turkey)	40	40	40	2	4, 1	Higher polytechnic	Bank employee	Egalitarian
Annelies	42	36	32	3	11, 9, 7	University	Bank employee	Egalitarian
Alisha (Surinam)	43	36	40	1	5	Secondary school	Project assistant	Egalitarian
Sheila (Chinese)	37	36	30	2	7, 5	Intermediate polytechnic	Personal assistant	Egalitarian
Rosa	32	36	–	2	12, 10	Intermediate polytechnic	Personal assistant	Egalitarian
39								
